# Altered distribution of the EphA4 kinase in hippocampal brain tissue of patients with Alzheimer’s disease correlates with pathology

**DOI:** 10.1186/s40478-014-0079-9

**Published:** 2014-07-16

**Authors:** Andrea FN Rosenberger, Annemieke JM Rozemuller, Wiesje M van der Flier, Philip Scheltens, Saskia M van der Vies, Jeroen JM Hoozemans

**Affiliations:** Alzheimer center & Department of Neurology, Neuroscience Campus Amsterdam, VU University Medical Center, De Boelelaan 1118, 1081 HZ Amsterdam, the Netherlands; Department of Pathology, Neuroscience Campus Amsterdam, VU University Medical Center, De Boelelaan 1117, 1081 HV Amsterdam, the Netherlands

**Keywords:** Alzheimer’s disease, EphA4 kinase, Synapse, Immunohistochemistry

## Abstract

Synaptic dysfunction occurs early in the progression of Alzheimer’s disease (AD) and correlates with memory decline. There is emerging evidence that deregulation of Erythropoietin-producing hepatocellular (Eph) receptor tyrosine kinases (RTK) signaling contributes to the aberrant synaptic functions associated with neurodegeneration. The Eph receptor A4 is highly expressed in human adult hippocampal brain tissue and was previously linked to cognitive impairment in a transgenic mouse model for AD. Whether EphA4 levels are altered in AD brain remains elusive. Therefore we investigated the protein levels and localization of EphA4 in human hippocampus derived from AD (n = 29) as well as non-demented control cases (n = 19).

The total EphA4 protein levels were not changed in AD patients compared to control cases. However, immunohistochemical localization of EphA4 revealed an altered distribution in AD compared to control hippocampus. EphA4 immunoreactivity was observed in plaque-like structures in AD cases. Double-labelling with phosphorylated tau and amyloid beta indicates that EphA4 co-localizes with neuritic plaques in AD. This altered distribution pattern was observed at early stages (Braak stage II) and correlates with the hallmarks of AD pathology suggesting a reduced availability of EphA4 that is likely to contribute to synaptic dysfunction that occurs early in AD.

**Electronic supplementary material:**

The online version of this article (doi:10.1186/s40478-014-0079-9) contains supplementary material, which is available to authorized users.

## Introduction

Alzheimer’s disease (AD) is the most common neurodegenerative disorder and has an increasing effect on our ageing population. Pathological hallmarks of AD are extracellular amyloid beta (Aβ) deposits and intracellular accumulation of hyper-phosphorylated tau protein leading to the formation of neurofibrillary tangles (NFTs) [1]. In addition, progressive synaptic dysfunction is thought to occur in early stages of the disease and has been found to correlate closely with cognitive deficits observed in patients with AD [2–4]. There is emerging evidence that the erythropoietin-producing hepatocellular (Eph) receptors and their ligands, the so-called ephrins, are involved in aberrant synaptic functions associated with cognitive impairment in AD [5]. Eph/ephrin signaling is required for a wide range of biological processes both during embryogenesis and adult life and involves the Eph receptors which form the largest of the 20 subfamilies of human receptor kinases. Eph/ephrin signaling plays a role not only during synapse formation and maturation and synaptic plasticity [6–8] in the brain but also in directing cell positioning and migration, axon guidance [9,10], control of tissue morphogenesis, patterning, tumour invasion and metastasis, immune function [11,12], haematopoiesis and blood clotting [13] and tissue repair and maintenance.

Eph receptors and their ligands are exclusively membrane-bound and hence cell-cell contact is required for activation of the kinase through oligomerisation and transphosphorylation [14]. EphA4 is the Eph receptor family member that is most highly expressed in the adult hippocampus where it plays a role in adult synaptic plasticity and learning [15,16]. The EphA4 kinase is pre- and post-synaptically expressed on dendritic spines of pyramidal neurons and axon terminals [17]. Emerging evidence supports a critical role for EphA4/ephrin A3 signaling in the regulation of spine morphology in the hippocampus. Activation of EphA4 upon binding to its glia-derived ligand ephrin A3 was found to induce spine retraction and to trigger the reduction of dendritic spines and synaptic proteins, whereas inhibiting those interactions led to distorted spine shape and organization in the murine hippocampus. These findings suggest an essential role for EphA4 in the elimination of excitatory synapses [18–20].

Two major forms of Aβ coexist in the brain: a shorter form with 40 amino acid residues and a longer form with 42 amino acids. The longer form is extremely toxic and can self-aggregate to form oligomers (amyloid beta oligomers, AβOs). Increased levels of EphA4 in cultured neurons and synaptoneurosomes was reported to be crucially involved in synaptic damage induced by AβOs [21]. Interestingly, reduced expression of the EphA4 receptor has been linked to cognitive impairment in a transgenic mouse model for AD overexpressing the human amyloid beta precursor protein (APP) [22]. Loss of synapses is an early event in AD pathogenesis. It has therefore been suggested that changes in hippocampal EphA4 signaling might precede the onset of memory decline in AD. Whether EphA4 levels and activation are altered in human AD brain is not known.

In the present study we are the first group to report the involvement of EphA4 in AD pathology. We have investigated EphA4 expression levels and localization in human brain tissue of patients with AD and non-demented controls. An association of EphA4 with the hallmarks of AD was investigated using sequential single stainings and double-labelling with phosphorylated tau and amyloid beta.

## Material and methods

### Case selection

Human brain tissue was obtained from the Netherlands Brain Bank (NBB, Amsterdam, The Netherlands). Prior to death, all donors gave written informed consent according to the Declaration of Helsinki for the use of their brain tissue and medical records for research purposes. This work was approved by the ethics committee of the NBB. Dementia status at death was determined on the basis of clinical information available during the last year of life. Neuropathological diagnosis was performed using histochemical analysis of formalin-fixed, paraffin-embedded tissue from different parts of the brain, including the frontal cortex (F2), temporal pole cortex, parietal cortex (superior and inferior lobule), occipital pole cortex and the hippocampus (essentially CA1 and entorhinal area of the parahippocampal gyrus) with routine stainings (hematoxilin and eosin, periodic acid Schiff-Luxol fast blue). Hippocampus and cortical areas were also stained with methenamine silver [23] and using the Bodian staining. Immunohistochemistry was performed using antibodies raised against hyperphosphorylated tau (AT8) and Abeta. Staging of AD pathology was evaluated according to modified assessment of Braak and Alafuzoff [24]. Cases with and without clinical neurological disease diagnosis were processed identically. Patients with co-morbidities like Parkinson’s disease or Lewy-body disease were excluded from the study. Age, sex, clinical diagnosis and Braak score for neurofibrillary tangles (NFTs) of all cases used in study are listed in Table 1. Post mortem delay of all cases was between 2 ½ and 9 hours. In total, 29 patients with AD and 19 controls were included (see Table 1), with an average age at death of 84 years (range 57 to 100 years old); of these, 22 (45.8%) were men.

**Table 1:** **Cases used in this study**

**Case no.**	**Pathological diagnosis**	**Braak stage**	**Braak (amyloid)**	**Sex**	**Age**	**PMD**
Immunohistochemistry						
1	CTRL	1	C	M	77	07:30
2	CTRL	1	B	F	77	02:55
3	CTRL	1	B	F	83	04:05
4	CTRL	1	B	F	85	07:05
5	CTRL	1	B	M	80	03:18
6	CTRL	1	0	F	81	04:25
7	CTRL	2	0	F	87	05:05
8	CTRL	2	0	F	76	05:45
9	CTRL	2	0	F	93	05:50
10	CTRL	2	B	F	89	06:25
11	CTRL	2	B	M	79	09:00
12	CTRL	2	0	M	81	07:55
13	CTRL	3	C	F	89	15:40
14	AD	3	B	M	84	05:20
15	AD	3	A	F	98	06:05
16	AD	3	B	F	96	04:00
17	AD	3	C	M	78	05:10
18	AD	3	C	F	82	06:05
19	AD	4	C	F	84	04:50
20	AD	4	C	M	88	05:00
21	AD	4	C	F	87	06:40
22	AD	4	C	F	100	05:15
23	AD	4	C	M	77	05:00
24	AD	5	C	F	84	04:30
25	AD	5	C	F	77	03:45
26	AD	5	C	F	78	08:25
27	AD	5	C	M	77	05:39
28	AD	5	C	M	86	06:15
29	AD	5	B	M	69	07:10
30	AD	6	C	M	62	06:45
31	AD	6	C	F	66	05:20
32	AD	6	C	F	62	-
33	AD	6	C	M	57	07:40
34	AD	6	C	F	84	04:50
35	AD	6	C	M	65	06:00
Double stainings						
36	AD	4	C	F	93	02:30
37	AD	5	C	M	93	04:30
38	AD	5	C	F	81	-
39	AD	6	C	M	74	05:35
40	CTRL	1	0	M	96	06:30
41	CTRL	1	A	F	94	04:05
42	CTRL	2	A	M	88	04:43
43	CTRL	1	B	F	77	02:55
Additional frozen tissue lysates						
44	AD	4	C	M	84	03:40
45	AD	4	C	M	81	04:50
46	AD	6	C	M	57	03:50
47	CTRL	1	0	M	58	05:15
48	CTRL	1	B	F	83	05:30

CTRL, control case; AD, Alzheimer’s disease case; PMD, post mortem delay; F, female; M, male.

### Western Blotting

Frozen hippocampal tissue slides of patients in all Braak stages (I-VI, n = 29) were cut and lysed with M-PER (pH 7.6, Thermo Scientific) containing protease and phosphatase inhibitors (Roche). After incubation for 30 min on ice and subsequent centrifugation (2 × 10 min, 4°C, 15682 × g), protein concentrations of the supernatants were determined with the standard Bradford Lowry Assay (Bio-Rad Protein Assay).

**Figure 1:**
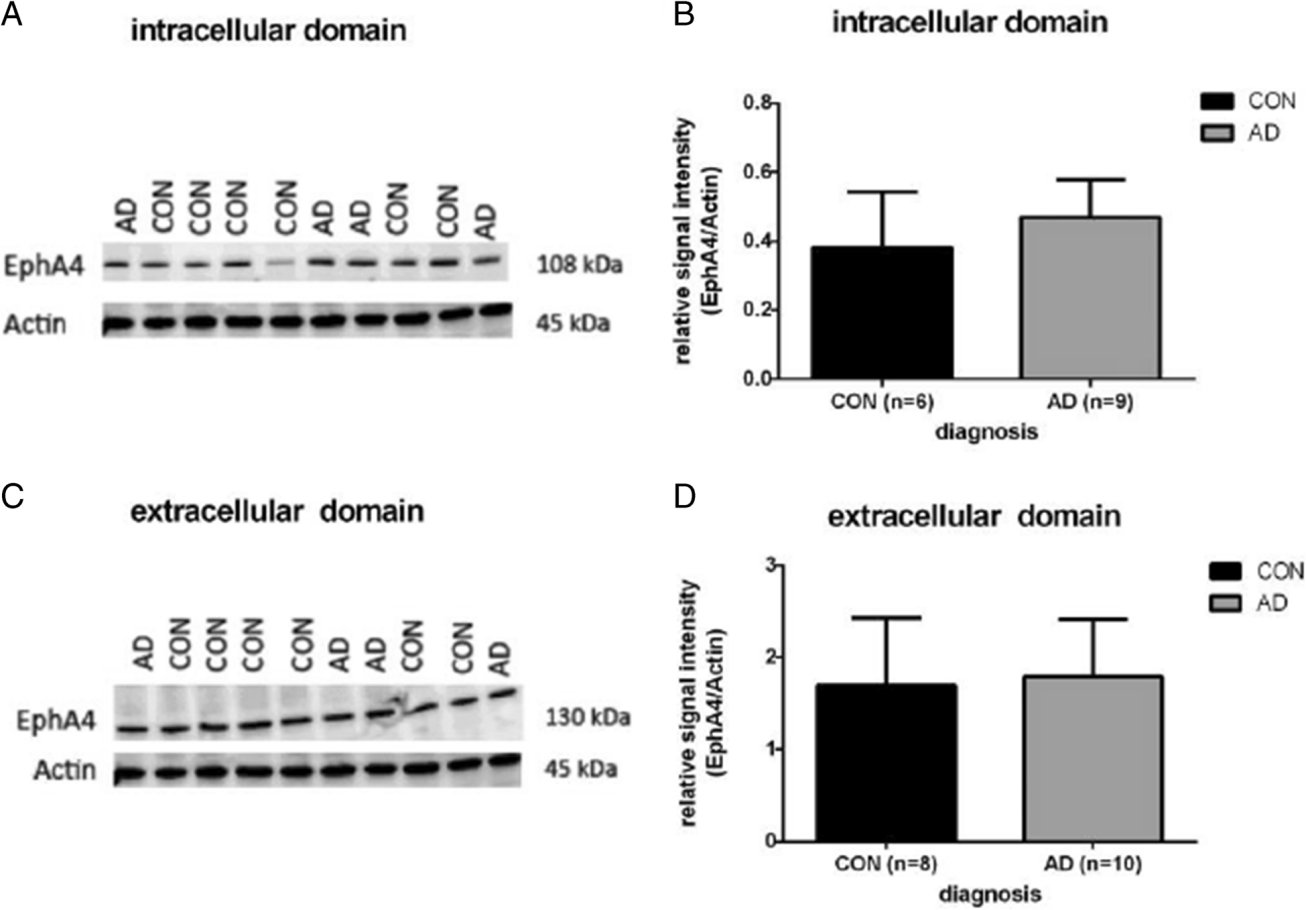
**Detection of EphA4 immunoreactivity in hippocampal brain tissue lysates of AD and non-demented controls.** Western blot analysis of Alzheimer’s disease **(AD)** and control (CON) cases for EphA4 with rabbit polyclonal antibody (aa 890–904, intracellular domain). **B)** Relative signal intensities (EphA4/Actin) of single cases per Braak stage (error bars indicate standard deviation) **C)** Western blot analysis for EphA4 mouse monoclonal antibody (aa 379–472, extracellular domain). **D)** Mean relative signal intensities with standard deviation.

Protein lysates were re-suspended in sample buffer (Thermo Scientific) and heated for 5 minutes at 95°C. Proteins were resolved by SDS-PAGE using 8–16% gradient polyacrylamide gels (mini-PROTEAN®TGX™, Bio-Rad) in running buffer (25 mM Tris, 192 mM glycine, 0.1% SDS, pH 8.3, Bio-Rad) and electrophoretically transferred onto a nitrocellulose membrane (0.2 μm; Whatman, Protran™) in transfer buffer (25 mM Tris, 192 mM glycine, 20% methanol, Bio-Rad). Membranes were blocked for 1 hour in Tris-buffered saline (50 mM Tris pH 7.5, 0.15 M NaCl, 0.1% Tween-20) containing 5% BSA (Roche Diagnostics) and incubated over night with primary anti-EphA4 antibody (1:500). A mouse monoclonal antibody directed at the extracellular (c-terminal) domain of the Ephrin-A4 receptor (amino acids 379–472; BD Transduction Laboratories) and a rabbit polyclonal EphA4 antibody directed at the intracellular domain (amino acids 890–904; Abcam) were utilised. Incubation with a secondary antibody linked to horseradish peroxidase ([HRP]-anti-rabbit IgG or HRP-anti-mouse IgG, 1:1000, DAKO) for 1 hour followed. Immunoreactive bands were detected with an enhanced chemiluminescence reagent (ECL Plus, GE Healthcare). Actin (mouse anti-actin AC-40, Sigma) was used as a loading control. The intensity of the resulting protein bands was quantified using MacBiophotonics ImageJ (version 1.47 k). Data is expressed as relative signal intensities (EphA4/Actin) of the individual samples (Figure 1). Statistical analyses were performed by t-test analysis for independent samples using GraphPad Prism version 6.0 (San Diego, CA). Furthermore, whole protein extracts were analysed by Immuno-blot analysis with SDS-PAGE using precast stainfree gradient gels (Bio-Rad; Additional file 1: Figure S6). After UV activation of the gel, proteins were visualised and relative protein amounts were determined and used for normalization. Recombinant proteins EphA1 (75 kDa, aa 569–976; Pro Quinase), EphA4 (72 kDa, aa 586–986; Carna Biosciences) and EphB2 (73 kDa, aa 581–987; Carna Biosciences) were used to proof specificity of the rabbit polyclonal EphA4 antibody. The EphA4 mouse antibody binds extracellularly (aa 379–472) which is outside of the binding domain for recombinant EphA4, therefore only the polyclonal rabbit antibody is shown (Additional file 2: Figure S8).

### Immunohistochemistry

**Figure 2:**
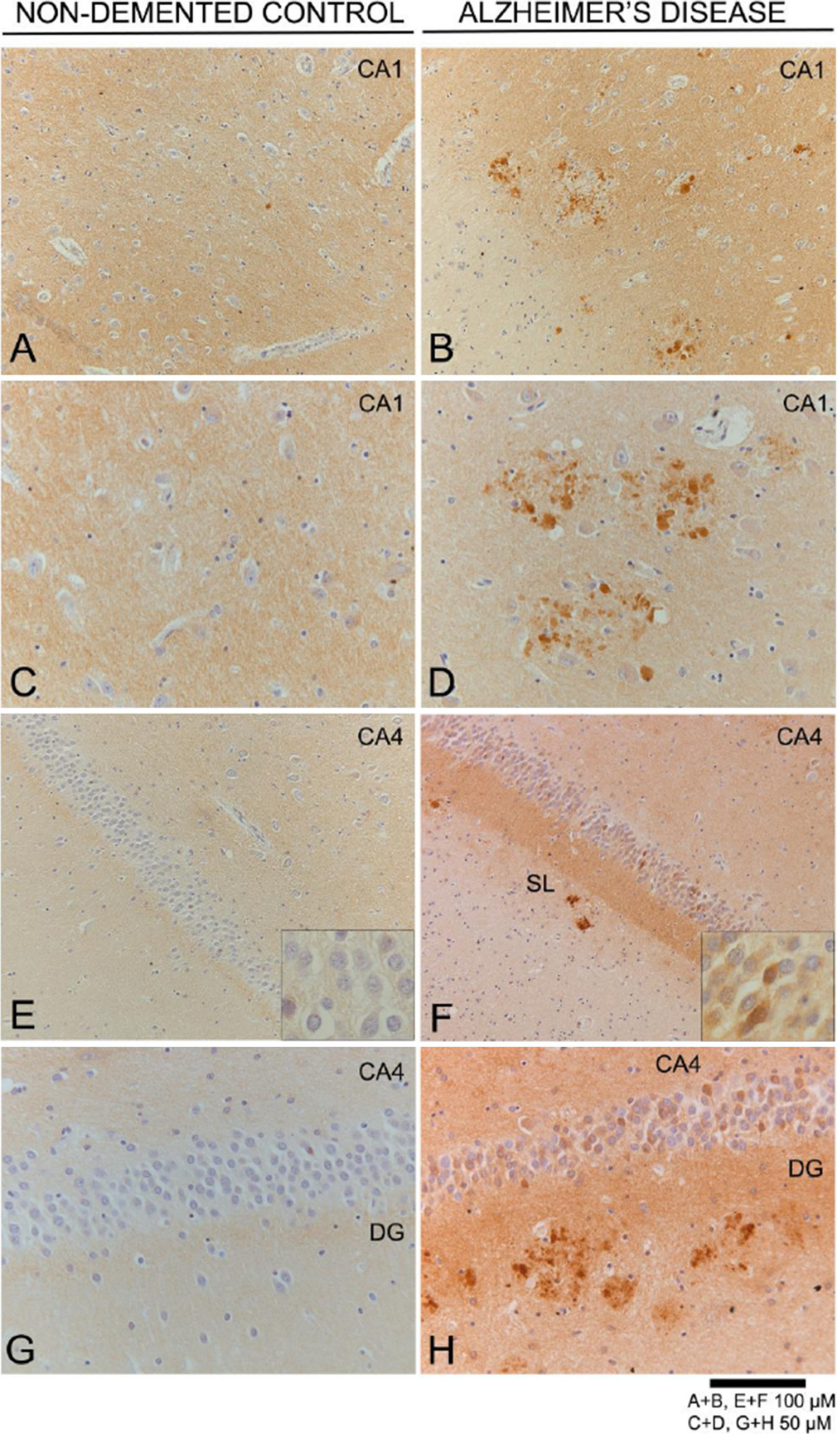
**Detection of EphA4 immunoreactivity in non-demented controls and AD hippocampus.** Immunohistochemical detection of EphA4 (DAB i.e. brown colour) in the CA1 region of **A)** non-demented control (Braak 2 O, #7 in Table 1) and **B)** AD (Braak 6 C, case #32 in Table 1) and with 2x higher magnification **C)** and **D)** respectively. EphA4 immunoreactivity in the dentate gyrus (DG) and stratum lacunosum (SL) in **E)** control (Braak 1 B, #5 in Table 1) and **F)** AD (Braak 6 C, #30 in Table 1) with 2x higher magnification in **G)** and **H)** respectively. Inserts in **E)** and **F)** are 2x higher magnifications of neurons of the granular layer (GL). The first panel **A-D** shows the CA1 region, whereas the second panel **E-H** are images from the CA4 and granular layer. All sections were counterstained with hematoxylin. Scale bar 100 μm for A + B, E + F and 50 μm for C + D, G + H.

**Figure 3:**
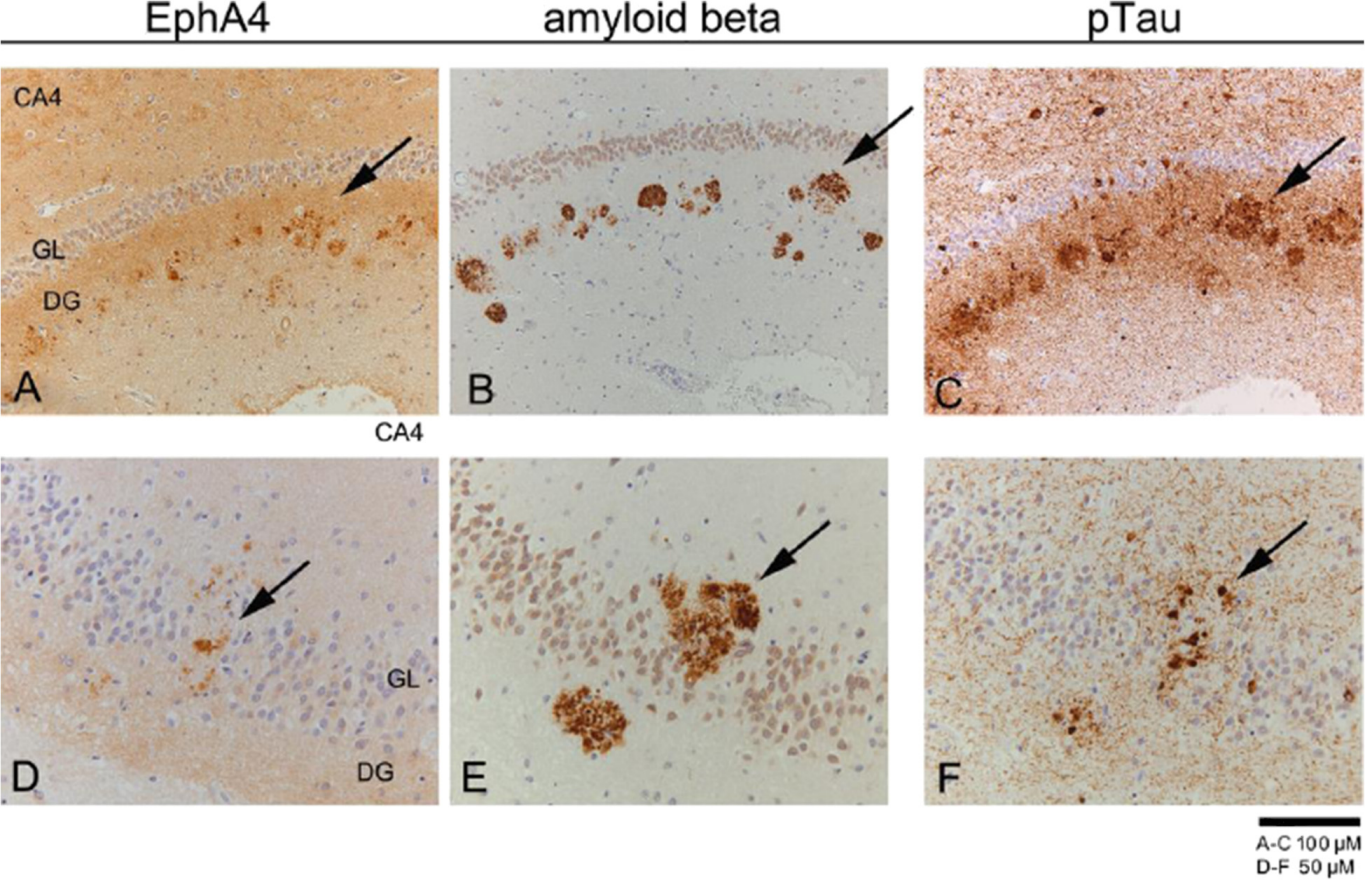
**Detection of EphA4, Aβ and pTau in consecutive brain slides of AD hippocampus.** Immunohistochemical detection of **A)** EphA4 (DAB i.e. brown colour), **B)** Aβ and **C)** pTau in the dentate gyrus (DG) and stratum lacunosum (SL) of consecutive brain slides in AD (Braak 6 C, case #30 in Table 1) indicated with the vessel in the right lower corner. Arrows are pointing out the same plaques. A 2x higher magnification is shown for EphA4, Aβ and pTau in **D)**, **E)** and **F)** respectively in consequent brain slides for a different patients with AD (Braak 6 C, case #31 in Table 1). All sections were counterstained with hematoxylin. Scale bar 100 μm for **A-C** and 50 μm for **D-F**.

For single immunohistochemical analysis, formalin fixed (4%, 24 hours) paraffin embedded 5 μm thick sequential sections were mounted on coated glass slides (Menzel Gläser Superfrost PLUS, Thermo Scientific, Braunschweig Germany), dried over night at 37°C and deparaffinised. In order to block the endogenous peroxidase, the sections were incubated in 0.3% (v/v) H_2_O_2_ in methanol (100%) for 30 min at RT. Sections were boiled in 10 mM sodium citrate buffer (pH 6.0) for 10 minutes for antigen retrieval. They were incubated over night with the primary antibody at room temperature. Mouse monoclonal anti-EphA4 [25–27] (1:200, BD Transduction Laboratories™); mouse monoclonal anti-phospho-tau [28] (AT8 1:200, Pierce Biotechnology, Rockford IL, USA); mouse monoclonal anti-Aβ (IC16 1:500, Prof. C. Korth, Heinrich Heine University Düsseldorf, Germany) and mouse monoclonal anti-Aβ 1–17 (VU17 [29] 1:1000, Dr. R. Veerhuis, VU University Medical Center, Amsterdam, NL) were used. As a second step, envision mouse/rabbit HRP (DAKO) was used. Sections were stained using the EnVision method (incubation with Dako REAL™ EnVison™ HRP rabbit/mouse antibody for 30 min). Subsequently, colour was developed using 3,3-diaminobenzidine (EV-DAB; DAKO) as chromogen. Sections were counterstained with hematoxylin and mounted using Quick-D mounting medium (Klinipath B.V., Duiven, The Netherlands) (Figures 2 and 3). Between all incubation steps, sections were washed extensively with phosphate buffered saline (PBS, pH 7.4), which also served as a negative control by omission of primary antibodies.

### Double immunohistochemistry: EphA4 with either Aβ or pTau

**Figure 4:**
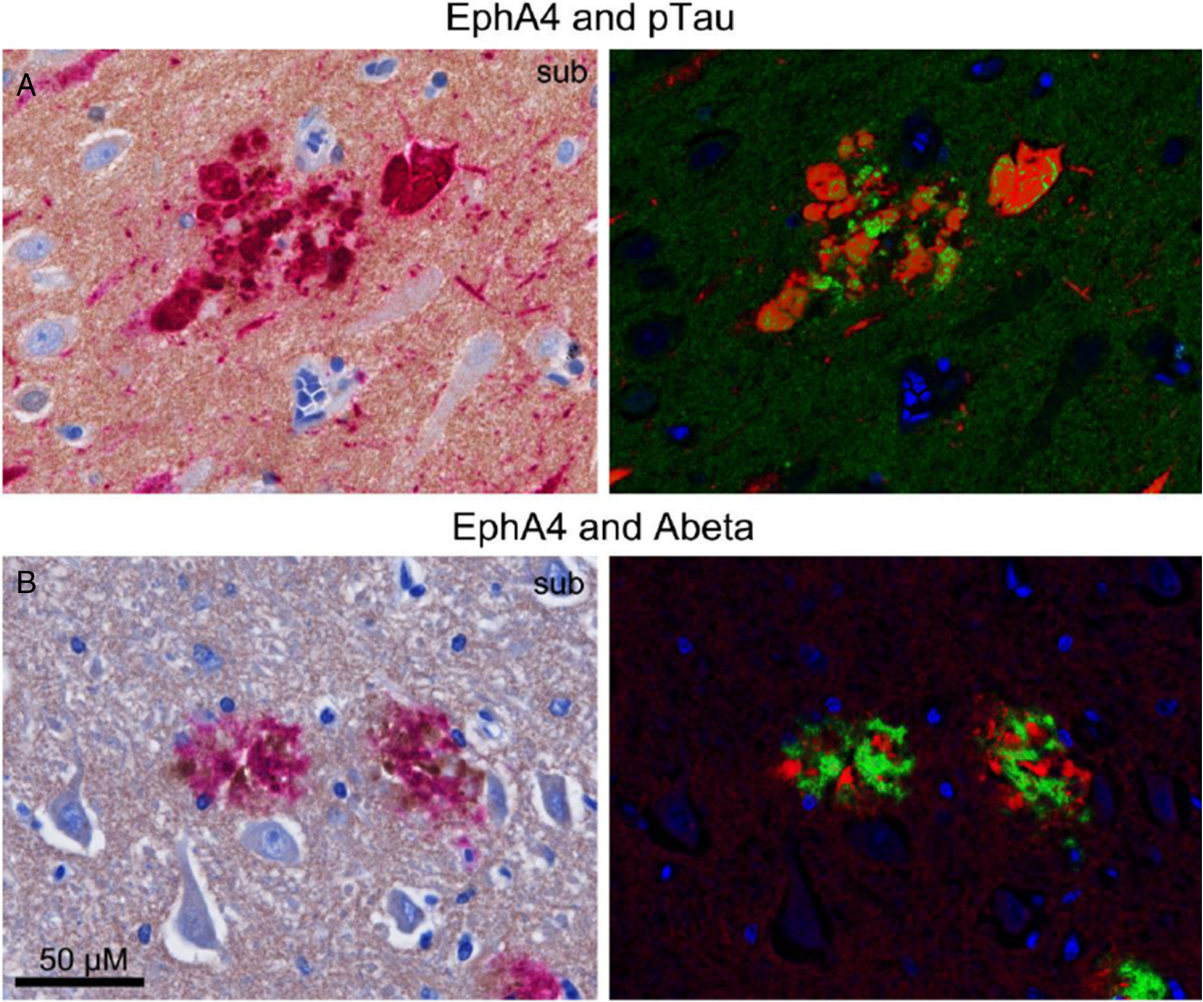
**Double-stainings of EphA4 with Aβ and pTau.** Double-stainings of EphA4 (DAB i.e. brown colour) with **A)** pTau (AT8, LPR i.e. pink colour) in a hippocampal area of a patient with severe AD (Braak 6, #39 in Table 1) and **B)** Aβ (VU-17, LPR i.e. pink colour). Spectral images on the right side (Aβ or pTau in red; EphA4 in green; nuclei in blue). Sections were counterstained with hematoxylin. Scale bar is 50 μm.

To determine the co-localization of EphA4 with amyloid beta and phosphorylated tau double immunohistochemistry was performed. Hippocampal brain slices were pre-incubated with serum-free protein blocking (SFPB, DAKO) for 10 min and subsequently incubated with mouse monoclonal anti-EphA4 [25–27] (1:200, IgG1, BD Transduction Laboratories™) for 1 hour. After washing with PBS, slices were incubated with EnVision solution (goat anti-rabbit HRP, undiluted, DAKO) for 30 minutes. Colour was developed using DAB as chromogen. Sections were treated with 10 mM sodium citrate buffer pH 6.0 heated at 180 Watt for 10 minutes. After pre-incubation with SFPB for 10 minutes, sections were incubated with anti-Aβ VU 1–17 antibody [29] (1:1000, IgG2a, VU medical center) or anti-pTau AT8 antibody (dilution 1:200, IgG1) for 1 hour. Sections were washed with PBS and incubated with either GαMIgG2a^HRP^ (dilution 1:100, Southern Biotech) for the detection of VU 1–17 or GαMIgG1^AF^ (dilution 1:100, Southern Biotech) for the detection of AT8 for 1 hour at RT. Colour was developed using Liquid Permanent Red (LPR, DAKO) as chromogen. Slices were counterstained with hematoxylin and mounted using Aquamount (BDH Laboratories Supplies). The Nuance™ spectral imaging system (CRi, Woburn, MA) was used for the analysis of double stained specimens. Spectral imaging data cubes were taken from 460–660 nm at 10 nm intervals and analyzed with the Nuance™ software. Spectral libraries of single-brown (DAB), single-red (LPR) and hematoxylin were obtained from control slides. The resulting library was applied to the double stained slides and the different reaction products were then spectrally unmixed into individual black-and-white images, representing the localization of each of the reaction products, and reverted to fluorescence-like images composed of pseudo-colours using the Nuance™ software (Figure 4).

### Analysis and quantification of EphA4 immunostaining

Presence of EphA4 in hippocampal subregions CA1 and subiculum were analysed in sections immunostained with EphA4 (BD Transduction Laboratories) by an observer blinded to the clinical and pathological diagnosis. Microscopic fields (n = 4, magnification 100x) were analysed (Table 2). The number of EphA4 depositions was scored as follows: 0, none; 1, one to ten depositions; 2, eleven to twenty depositions; 3, more than twenty. The presence of Aβ plaques (plaque severity) was quantified in a similar manner (0, none; 1, occasional plaque; 2, several plaques scattered throughout the field; 3, abundant presence of plaques). The presence of pTau-tangles was scored: 0, none; 1, mild (occasional immunoreactivity); 2, moderate (scattered throughout the field); 3, severe (abundant presence of plaques). Additional file 3: Figure S7 shows examples for scoring of EphA4, Aβ and pTau immunoreactivity.

**Table 2:** **Scoring of EphA4, A**β **plaques, pTau plaques and pTau tangles**

**Case no.**	**Braak**	**Braak (amyloid)**	**EPHA4 plaques**	**pTau plaques**	**pTau tangles**	**Aβ** **plaques**
1	1	C	+	++	+	++
2	1	B	0	0	0	+++
3	1	B	+	0	+	+++
4	1	B	+	0	0	+
5	1	B	0	0	+	++
6	1	0	0	0	0	0
7	2	0	+	0	++	0
8	2	0	0	0	+++	0
9	2	0	+++	0	0	0
10	2	B	+	+++	+	+++
11	2	B	++	+	++	++
12	2	0	+	0	++	+
13	3	C	+++	+++	+++	+++
14	3	B	0	+	+	+++
15	3	A	+	0	++	+
16	3	B	+	++	++	+
17	3	C	+	++	+++	+++
18	3	C	+	0	+	+++
19	4	C	+++	++	+++	+++
20	4	C	+	+++	++	++
21	4	C	++	0	++	+++
22	4	C	+	+	+++	+++
23	4	C	+++	+	+++	+++
24	5	C	+++	++	+++	+++
25	5	C	+++	+++	+++	++
26	5	C	+++	+++	++	+++
27	5	C	+	+++	++	+++
28	5	C	+	0	+++	+++
29	5	B	+++	+++	+	+++
30	6	C	+++	+++	+++	+++
31	6	C	+++	+	+++	+++
32	6	C	+++	++	+++	+++
33	6	C	0	+++	+++	+++
34	6	C	+++	+++	+++	+++
35	6	C	+	+++	++	+++

### Statistical analysis

**Figure 5:**
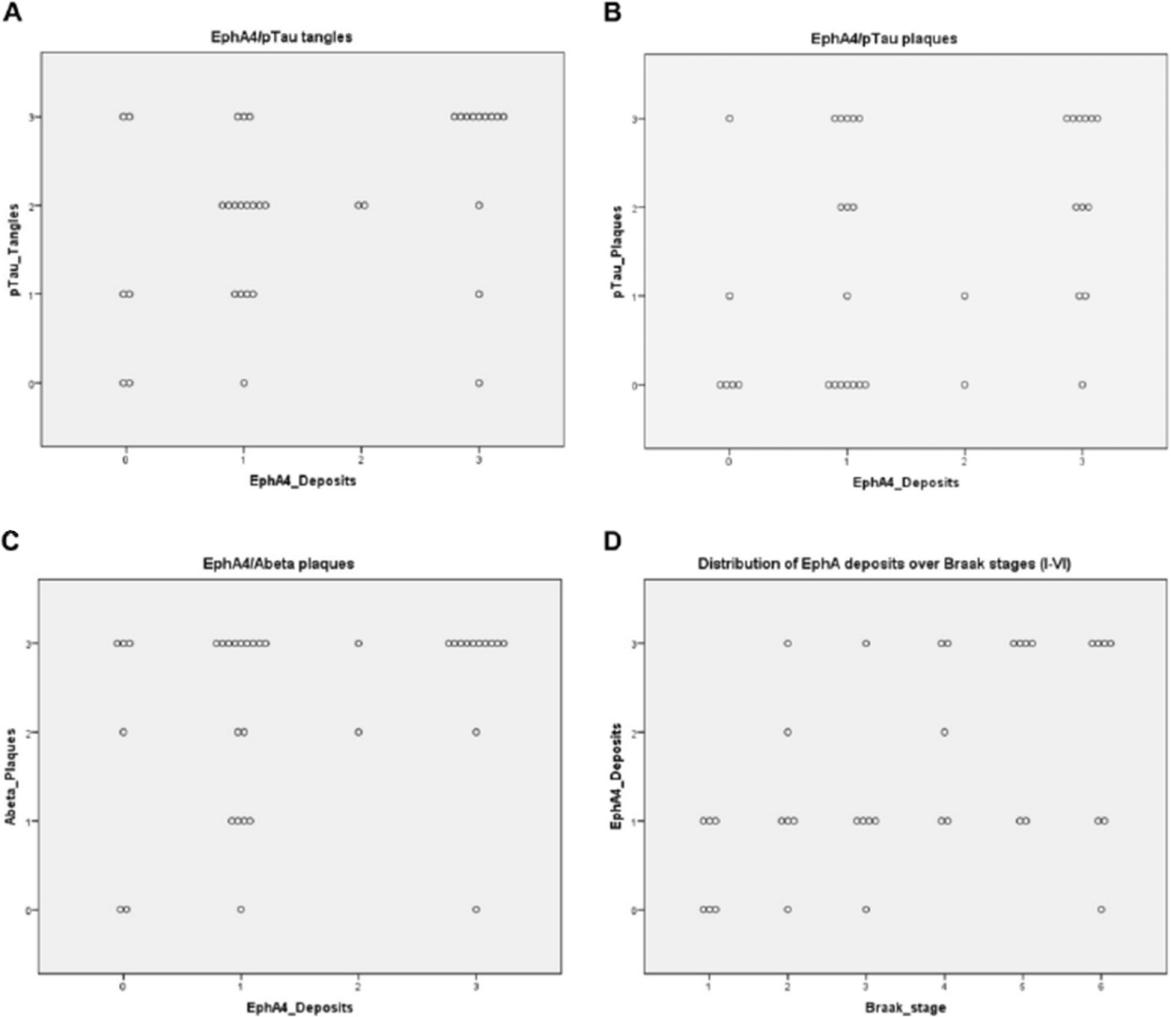
**Pairwise correlation analysis of EphA4, pTau plaques and tangles and Aβ** **plaques.** Correlation of EphA4 deposits with **A)** pTau tangles, **B)** pTau plaques and **C)** Abeta plaques. **D)** Distribution of EphA4 deposits over Braak stages (I-VI). E) Table with the corresponding correlation coefficients (Spearman’s rho and Kendall’s tau) as well as p-values. Correlations are considered significant when p < .05.

Pairwise correlation analyses for non-parametric ordinal data were conducted using IBM SPSS Statistics 22.0 (Figure 5A-D). Spearman’s correlation coefficients and Kendall’s tau coefficients were calculated. Correlations were considered significant in the 95% confidence interval when p < 0.05 (Table 3).

**Table 3:** **Correlation coefficients for pairwise comparisons of EphA4 with the hallmarks of AD and Braak stage (according to Figure** 5 **)**

		**Kendall’s tau [τ]**	**Spearman’s rho [r**_**s**_ **]**	**significance [p < .05]**
A	EphA4/pTau tangles	.393, p = .007*	.420, p = .011*	significant
B	EphA4/pTau plaques	.329, p = .025*	.378, p = .023*	significant
C	EphA4/Abeta plaques	.259, p = .084	.282, p = −096	not significant
D	EphA4/Braak stage	.421, p = .003*	.486, p = .003*	significant

## Results

In the present study we report on the involvement of EphA4 in human AD pathology. We determined the levels of EphA4 in hippocampal brain tissue of AD and non-demented control patients and found an association with the two hallmarks of AD (pTau and Abeta) using immunohistochemical analysis.

### Expression levels of EphA4 in the human hippocampus at different stages of AD

In order to investigate protein levels of EphA4 we analysed frozen hippocampal brain tissue lysates of AD patients (n = 18) and non-demented controls (n = 11) by Western Blotting. Using two different EphA4 antibodies i.e. a rabbit polyclonal antibody raised against the intracellular part of the EphA4 and a mouse monoclonal antibody to detect the extracellular domain of EphA4. The rabbit antibody detected a protein (fragment) of approx. 108 kDa whereas the mouse antibody showed a band that correlates to a molecular weight of 130 kDa (Figure 1 A,C). Actin was used as a loading control (molecular weight is ~ 45 kDa) and to determine relative signal intensities for EphA4 (Figure 1B,D). We observed no significant difference in the levels of the intracellular and extracellular domain of EphA4 between AD cases and controls (Figure 1). We analysed the levels of EphA4 at different stages of AD and found no significant differences (results not shown). Since EphA4 is an integral membrane protein we analysed whole tissue lysates (containing soluble and insoluble fraction). No difference in average levels was observed between AD and control hippocampus (see Additional file 1: Figure S6). In conclusion we observed no differences in EphA4 levels in hippocampal tissue at different stages of AD. Recombinant proteins EphA1, EphA4 and EphB2 were used to prove specificity of the rabbit polyclonal EphA4 antibody. The mouse anti-EphA4 antibody detects EphA4 at aa 379–472. This sequence is not present in recombinant EphA4 which represents a shorter fragment of EphA4. Therefore mouse anti-EphA4 antibody is not able to detect recombinant EphA4 (Additional file 2: Figure S8). No bands were observed for recombinant EphA1 and EphB2 whereas a band for recombinant EphA4 was detected at 72 kDa for all three concentrations (1:50, 1:100, 1:200). This suggests highly specific binding of the rabbit EphA4 antibody.

### EphA4 localization in patients with Alzheimer’s disease

Since we observed no difference in the levels of EphA4 we wondered whether the localisation of EphA4 might change in AD versus non-demented controls. The mouse monoclonal antibody directed at the extracellular (c-terminal) domain of EphA4 was used for detection of the protein. In non-demented control cases (without AD pathology), a low intense immune-reactive signal of EphA4 was detected in the parenchyma. The signal was evenly distributed throughout the hippocampus (Figure 2A,C). In contrast, in AD cases EphA4 immuno-reactivity was observed in plaque-like structures (Figure 2B,D). Interestingly, EphA4 immuno staining of these plaque-like structures was observed more frequently in the higher Braak stages (V and VI), but some of the samples representing low stages (three in Braak I and 5 in Braak II) showed the same pattern. The EphA4-positive plaque-like structures showed considerable variations in shape and size (5–50 μm) and were detected in all subregions of the hippocampus.

Intense EphA4 immunostaining was observed in the cytoplasm of granular neurons in the dentate gyrus of AD but not in the dentate gyrus of non-demented control cases (Figure 2E,F). In non-demented controls, EphA4 immunoreactivity was found as a fine punctate line in the plexiform layers of the dentate gyrus (Figure 2G). In AD patients, EphA4-positivity was frequently decorating the dentate gyrus showing a brown band in the perforant path target zone of the outer molecular layer of the dentate gyrus, forming a frame around the granular layer (Figure 2F,H). This staining pattern was not observed in control cases. EphA4-positivity was not restricted to specific subareas of the hippocampus but was also found in and around neurons of the granular layer and in the stratum lacunosum. The presence of EphA4 immunoreactive plaque-like deposits in the CA regions of the hippocampus and entorhinal cortex in combination with the intense EphA4 immunostaining of the molecular layer was most prominent in demented individuals. Interestingly, EphA4 immunoreactive plaques were also observed in the hippocampi of non-demented controls (Braak II, see scoring Table 2) which points towards an early involvement of EphA4 in AD pathology.

### EphA4 partially co-localizes with pTau and neuritic Aβ plaques

The detection of EphA4 immunoreactivity plaque-like structures suggests a co-localization with neuritic plaques. In order to investigate the co-occurrence of EphA4 with neuritic plaques, adjacent brain tissue sections were stained for EphA4, pTau and Aβ (Figure 3). Consecutive brain slices were stained for Aβ, pTau and EphA4 and partial co-localization in the hippocampus of AD patients was revealed.

In order to corroborate the possible co-localization of EphA4 with either Aβ or pTau, double-immunostaining was performed. We found confirmation of partial co-localization of EphA4 with pTau and Aβ-immunoreactive plaques in AD patients (Figure 4A). EphA4-immunoreactivity was characteristically found at the ends of dystrophic neurites around central Aβ plaques (Figure 4B). EphA4 immunoreactivity is less intense compared to that of Aβ or pTau which could reflect differences in the antigen binding properties of the different antibodies. So EphA4 localization is clearly linked to the deposition of both Aβ and pTau.

### EphA4 depositions in the hippocampus of AD patients at different Braak stages

In order to investigate whether the number of EphA4 depositions increases with Braak stage and correlates with the hallmarks of AD, immunoreactivity for EphA4 was analysed in a new second cohort representing all Braak stages (I-VI) (Table 2). Adjacent tissue sections were stained and scored for EphA4 depositions, Aβ plaques, pTau-positive plaques and pTau tangles (see Additional file 3: Figure S7 for scoring example).

To examine the relationship between EphA4 and the hallmarks of AD, Spearman’s correlation [r_s_] and Kendall’s correlation [τ] analyses for non-parametric ordinal data were performed (Table 3). Spearman’s correlation coefficients are used for correlation analyses of non-parametric data sets. Kendall’s correlation coefficients are preferred for small sample sizes with a large number of tied ranks. The analyses revealed a significant correlation between EphA4 positive depositions and pTau-positive tangles (Figure 5A; r_s_ = .420, p < .05; τ = .393, p < .05) and between EphA4 and pTau-positive plaques (Figure 5B; r_s_ = .378, p < .05; τ = −.329, p < .05). No significant correlation was found between EphA4-load and Aβ plaques (Figure 5C; r_s_ = .282, ns; τ = .259, ns). However, the correlation between EphA4 deposits and Braak stage was evident (Figure 5D; r_s_ = .486, p < .05; τ = .421, p < .05). Table 3 summarizes the obtained correlation coefficients and p-values.

## Discussion

Synaptic loss is one of the major pathological hallmarks of AD. This is considered to be an early event in the pathogenesis of the disease. Synaptic failure correlates with cognitive decline and is observed in patients with mild cognitive impairment (MCI) and incipient AD [3]. The molecular mechanism of synaptic dysfunction in AD remains elusive.

The tyrosine kinase receptor EphA4 is essential for synaptic function as it is involved in dendritic spine morphogenesis, synapse formation and maturation [5]. EphA4 is highly expressed in the adult hippocampus, where it is known to play a role in adult synaptic plasticity and learning [16,30]. Of all cortical areas, the hippocampus appears to be most severely affected by the loss of synaptic proteins in AD (44 to 55%) [31]. Interestingly, analysis of synaptoneurosomes from AD patients revealed a ~2-fold increase in EphA4 mRNA [32], suggesting a role in synaptotoxicity for EphA4 Therefore we investigated EphA4 expression and localization in the hippocampus of patients at various Braak stages.

In this study the total EphA4 protein levels were similar in AD patients compared to control cases. However, immunohistochemical localization of EphA4 revealed an altered distribution in AD compared to control hippocampus. EphA4 partially co-localized with neuritic amyloid beta plaques. An aberrant function of EphA4 might be the underlying cause for the altered distribution of EphA4 in different Braak stages. Thus, EphA4 could be contributing to synaptic dysfunction which is considered an early event in AD.

So far, reports about changes in EphA4 protein levels in AD hippocampus have been contradictory. Simón et al. reported a reduction of EphA4 (20%) in hippocampal tissue of three patients with very mild cognitive deficits (Braak stage II and III) compared to three control subjects [33]. Matsui et al. showed that total protein levels of EphA4 in AD brains were not altered compared to controls. Furthermore they reported a decrease of membrane associated EphA4 (intracellular domain) in frontal lobes of AD cases while the amount of full-length EphA4 was unchanged [34]. In the present study we investigated a large cohort (n = 35) covering all Braak stages using two different antibodies directed at different epitopes of EphA4. EphA4 fragments with different molecular weights (108 kDa vs. 130 kDa) were detected. This discrepancy can be explained by the different binding sites of the used antibodies. Overall, we were not able to detect changes in EphA4 protein levels between control and AD groups and between different Braak stages when we used Western Blot analysis.

In contrast, at the immunohistochemical level we observed differences in the staining patterns of EphA4 when comparing control and AD cases. We showed that depositions of the EphA4 protein kinase are present in all subareas of the hippocampus in AD patients. The number of EphA4 deposits increases with Braak stage and those deposits partially co-localize with neuritic plaques and tangles. Moreover, EphA4 immunoreactive plaques are already present in Braak stage II which points towards an involvement of EphA4 in the early stages of AD pathology. The increased occurrence of EphA4 deposits with AD pathology in the absence of changes in total EphA4 protein levels indicate an altered distribution of EphA4. The important role of EphA4 in synaptic dysfunction, an early event in AD, has been reported [35]. Pathological changes happen in the brain even decades before the first clinical symptoms emerge. Therefore, it is likely that the aberrant EphA4 staining in part of the control cases (Braak stage II and III) poses an early event in AD pathogenesis and is therefore specific.

Mostly, investigations into AD related synaptic changes have focused on the toxic effects of Aβ. Like the amyloid precursor protein (APP), EphA4 is a substrate of γ-secretase [34]. The EphA4 intracellular domain (EICD) that remains after cleavage is known to enhance the formation of dendritic spines via activation of the Rac signaling pathway [34]. It has been suggested that aberrant γ-secretase activity followed by hindered cleavage of EphA4 results in reduced formation of dendritic spines and may be the major cause of synaptic failure in AD [36]. In addition, reduced EphA4 levels have been reported in whole-cell lysates of hAβPP_swe-ind_ mice and Tg2576 mice compared to non-transgenic mice [33]. Those hAβPP_swe-ind_ mice show amyloid-related pathology but no accumulation of pTau in tangles. These data strengthen the possible link between Aβ and aberrant EphA4 signaling.

The emerging view is that toxic amyloid-β oligomers (AβOs) are an important pathological factor in early neurodegenerative events in AD [37]. Two major forms of Aβ coexist in the brain: a shorter form with 40 amino acid residues and a longer form with 42 amino acids. The longer form is extremely toxic and can self-aggregate to form oligomers (amyloid beta oligomers, AβOs) [2]. Those oligomers accumulate into Aβ deposits in patients with AD. It has been reported recently that activation of the Abelson non-receptor tyrosine kinase c-Abl, a kinase downstream of EphA4, mediates synaptic loss and long term potentiation in dendritic spines of cultured rat hippocampal neurons. The co-localization of c-Abl with amyloid plaques, neurofibrillary tangles and granulovacuolar degeneration in the hippocamups and entorhinal cortex of AD patients has been reported in 2009 by Jing et al. [38]. AβOs activate the c-Abl kinase and thereby induce synaptic loss [21]. Concomitantly, EphA4 tyrosine phosphorylation is increased in these cultured neurons and in synaptoneurosomes exposed to AβOs. EphA4/c-Abl activation is a key-signaling event mediating the synaptic damage induced by AβOs. EphA4/c-Abl signaling could hence be a relevant pathway involved in the early cognitive decline observed in AD patients [21].

In human brain, a stronger relation between EphA4 and pTau positive plaques exists. In AD, c-Abl is detected in neurofibrillary tangles [38] and phosphorylates tau directly [39] and through activation of Cdk5 [40]. In the present study, we show a significant correlation between EphA4 positive depositions and pTau-positive plaques and an almost significant correlation with pTau-positive tangles in AD. Also Matsui et al. previously reported a correlation between the intracellular domain of EphA4 (EICD) and tau phosphorylation, although the correlation did not reach statistical significance. In contrast, the level of EICD did not correlate with the level of Aβ [34].

Tau may be involved in synaptic dysfunction in dementia [36], strengthening the association with EphA4 [5]. A key question remains whether the association with EphA4 is causally related to tau phosphorylation and aggregation. Either aberrant EphA4 signaling promotes phosphorylation of tau contributing to the formation of neurofibrillary tangles and neuronal dystrophy, or on the contrary, aggregating tau affects the subcellular distribution of key proteins involved in synaptic function such as the EphA4 receptor. So far several groups have reported that Eph/ephrin signaling up-regulates tau expression and phosphorylation [41,35]. When EphA4 is activated by ephrin A1 it recruits and phosphorylates cyclin-dependent kinase 5 (CDK5) [35]. CDK5 is a tau kinase and is increased in AD brain [42]. Increased CDK5 immunoreactivity is observed in pretangle neurons supporting its involvement in early stages of AD pathogenesis [43]. The significance of a dysregulation of CDK5 by EphA4 in pathological conditions remains elusive.

In our study, EphA4 depositions partially co-localize (~30%) with neuritic Aβ plaques in human hippocampal brain tissue. Manczak et al. recently reported a physical interaction between Aβ and phosphorylated Tau [44]. They found an abnormal interaction of oligomeric Aβ with pTau in neurons of post mortem brains from AD patients. This interaction may be involved in neuronal and synaptic damage, leading to cognitive decline in incipient AD patients [44]. Those findings may explain that EphA4 is related to both Aβ and pTau and suggests an underlying common pathway. Further studies are necessary to shine light on the relation of those proteins and the significance in the progression of AD.

Four recent large late-onset AD (LOAD) genome-wide association studies (GWAS) have identified EPHA1 as a genetic factor. Like EphA4, EphA1 is suggested to be important for synaptic function [12,45]. This supports the emerging evidence that the Eph receptors and their ligands, the ephrins, are involved in aberrant synaptic functions associated with cognitive impairment in AD [5]. In this study we show that EphA4 co-localizes with neuritic plaques in human brain tissue of patients with AD. EphA4 is associated with both Aβ and pTau. The altered distribution of EphA4 in AD hippocampus may reflect a decreased function of EphA4, which is likely to contribute to synaptic dysfunction that occurs in the early stages of AD. Pathological changes happen decades before the first clinical symptoms emerge explaining EphA4-positivity in early Braak stages (II and III, see Table 2).

## Additional files

Additional file 1: Figure S6. Example of EphA4 detection in whole tissue lysates by Western blot analysis. A) Western blots with whole tissue lysates of hippocampi of all Braak stages (0-VI). B) Box blot analysis of Western Blots for EphA4. Signal intensity for Braak 0-III and Braak IV-VI. Specificity p < 0.05. Additional file 2: Figure S8. Western Blot analysis of specificity of EphA4 (rabbit) antibody against recombinant EphA1 and EphB2. The rabbit polyclonal EphA4 antibody is specific for EphA4 (band at 72 kDa). No bands are observed for recombinant EphA1 and EphB2. Additional file 3: Figure S7. Example of scoring for quantification of EphA4, pTau and Aβ. For explanation of scoring see material and methods and Table 2.
