# Supplementary material for: Altered distribution of the EphA4 kinase in hippocampal brain tissue of patients with Alzheimer’s disease correlates with pathology
Source: Acta Neuropathol Commun. 2014 Jul 16;2:79. doi: 10.1186/s40478-014-0079-9 (PMC4149234; doi:10.1186/s40478-014-0079-9)
Supplement: Supplementary file 1 [file 40478_2014_79_MOESM1_ESM.doc]

Fig. 6

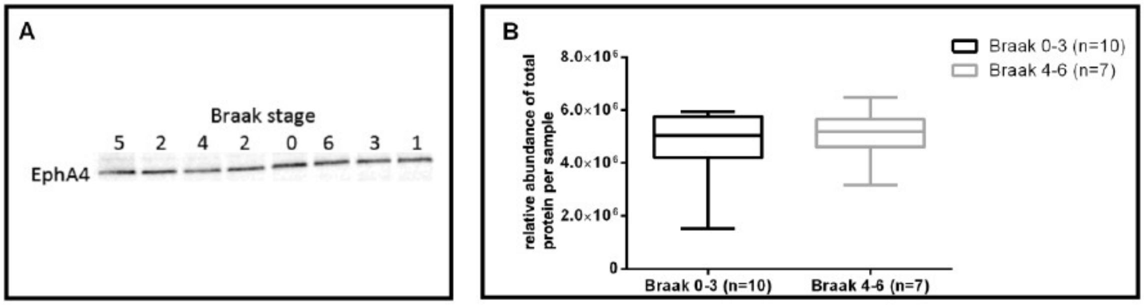
A
Braak stage
5 2 4 2 0 6 3 1
EphA4
B
Braak 0-3 (n=10)
Braak 4-6 (n=7)
8.0×10⁶
6.0×10⁶
4.0×10⁶
2.0×10⁶
0
relative abundance of total protein per sample
Braak 0-3 (n=10)
Braak 4-6 (n=7)
