# Supplementary material for: Altered distribution of the EphA4 kinase in hippocampal brain tissue of patients with Alzheimer’s disease correlates with pathology
Source: Acta Neuropathol Commun. 2014 Jul 16;2:79. doi: 10.1186/s40478-014-0079-9 (PMC4149234; doi:10.1186/s40478-014-0079-9)
Supplement: Supplementary file 2 [file 40478_2014_79_MOESM2_ESM.doc]

Fig. 8

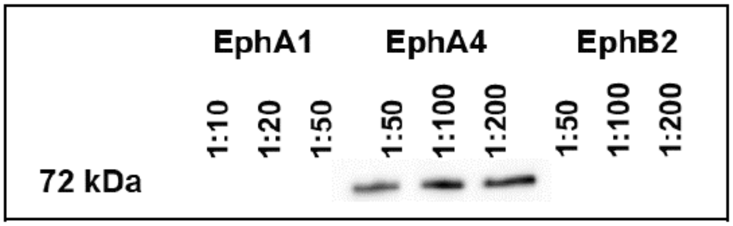
EphA1
EphA4
EphB2
1:10
1:20
1:50
1:50
1:100
1:200
1:50
1:100
1:200
72 kDa
