# Supplementary material for: Altered distribution of the EphA4 kinase in hippocampal brain tissue of patients with Alzheimer’s disease correlates with pathology
Source: Acta Neuropathol Commun. 2014 Jul 16;2:79. doi: 10.1186/s40478-014-0079-9 (PMC4149234; doi:10.1186/s40478-014-0079-9)
Supplement: Supplementary file 3 [file 40478_2014_79_MOESM3_ESM.doc]

Fig. 7

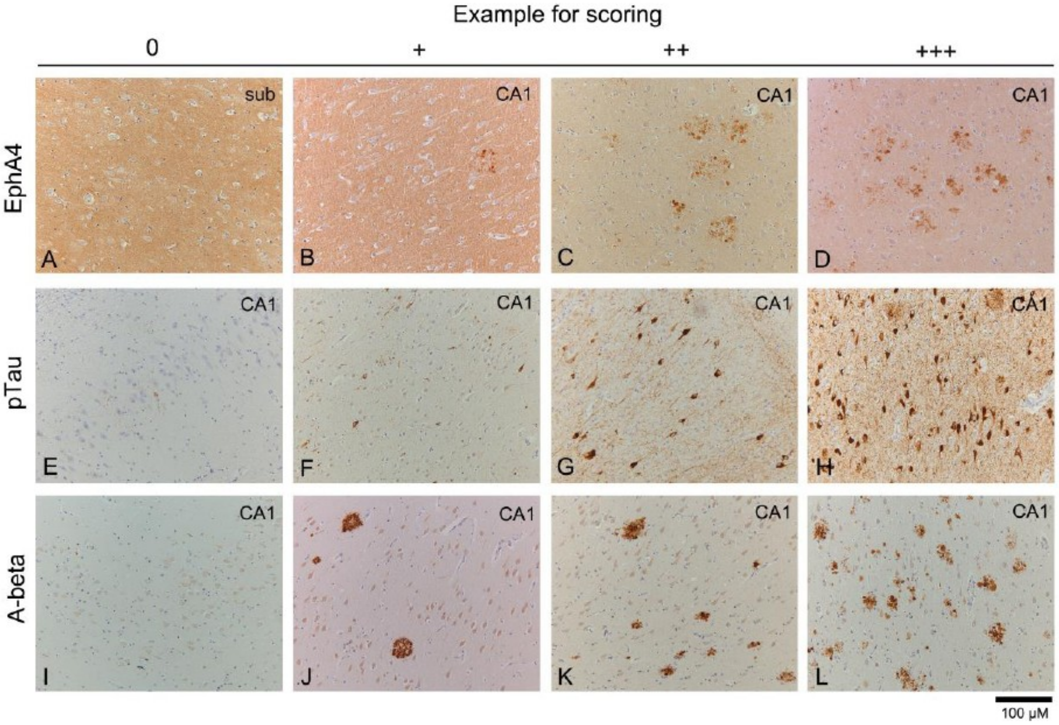

Example for scoring
0
+
++
+++
EphA4
pTau
A-beta
sub
CA1
A
B
C
D
E
F
G
H
I
J
K
L
100 μM
